# Evaluation of nitazoxanide in the treatment of experimental murine neurotoxoplasmosis

**DOI:** 10.1590/S1678-9946202466061

**Published:** 2024-10-11

**Authors:** Thaís Santos Anjo Reis, Victor da Silva Siqueira, Stéfanne Rodrigues Rezende Ferreira, Natália Domann, Benílton Alves Rodrigues, Amanda Cristina Corrêa Fleury, Isa Marianny Ferreira Nascimento Barbosa de Souza, Ludimila Paula Vaz Cardoso, Carla Silva Siqueira, Hanstter Hallison Alves Rezende

**Affiliations:** 1 Universidade Federal de Jataí, Instituto de Ciências da Saúde, Programa de Pós-Graduação em Ciências Aplicadas à Saúde, Jataí, Goiás, Brazil; 2 Universidade Federal de Jataí, Instituto de Ciências da Saúde, Curso de Biomedicina, Jataí, Goiás, Brazil

**Keywords:** Toxoplasma gondii, Neurotoxoplasmosis, Sulfadiazine, Pyrimethamine, Nitazoxanide, Treatment

## Abstract

Toxoplasmosis is a widespread zoonotic disease that poses significant public health concern globally, with neurotoxoplasmosis being a severe complication associated with high mortality rates. The standard therapy for neurotoxoplasmosis involves a combination of sulfadiazine and pyrimethamine, which, despite its efficacy, is often limited by adverse effects leading to treatment discontinuation. This study aimed to evaluate the *in vivo* efficacy of nitazoxanide in treating neurotoxoplasmosis in mice infected with the Me49 strain. The study comprised two groups: Group I, including subgroups of uninfected, infected and treated with saline, and infected and untreated mice; and Group II, comprising infected mice treated with nitazoxanide at 100 mg/kg/day, nitazoxanide at 150 mg/kg/day, and pyrimethamine combined with sulfadiazine. After 14 days of treatment, the mice were euthanized for organ collection. Histopathological examination of the brains revealed that the highest dose of nitazoxanide reduced parasitic load and cerebral hemorrhages. Biochemical and histopathological analyses of liver and kidney tissues demonstrated toxicological profiles comparable to pyrimethamine and sulfadiazine. However, despite showing efficacy and similar toxicity levels, nitazoxanide treatment was less effective regimen in controlling neurotoxoplasmosis in this experimental model compared to the pyrimethamine and sulfadiazine. Thus, while nitazoxanide presents potential in neurotoxoplasmosis treatment, pyrimethamine combined with sulfadiazine remains the preferred therapeutic choice based on better efficacy observed in this study.

## INTRODUCTION

Toxoplasmosis is caused by the obligate intracellular protozoan *Toxoplasma gondii*, which can infect and multiply in most host cells. *T. gondii* infects both warm- and cold-blooded animals such as mammals, reptiles, and birds, with humans serving as intermediate hosts and felines acting as definitive hosts^1^.

Toxoplasmosis usually manifests asymptomatically in immunocompetent individuals, persisting as a chronic infection. However, in immunocompromised patients, such as transplant recipients, those undergoing chemotherapy, or people living with HIV/AIDS (PLHIV), neurotoxoplasmosis (NTX) frequently develops. NTX is characterized by the reactivation of brain cysts that were previously in a latent state^2,3^.

The first-line treatment for NTX involves a combination of pyrimethamine, sulfadiazine, and folinic acid. Folinic acid, or leucovorin, is added to reduce the likelihood of hematological toxicities associated with pyrimethamine. Pyrimethamine is considered standard chemotherapy for NTX treatment due to its efficient penetration into the cerebral parenchyma, even in the absence of inflammation^4^.

Despite being the standard treatment for NTX, this therapeutic approach shows various side effects and high toxicity, requiring changes in the treatment regimen^5^. Another limitation is that the medications administered have remained the same since the 1950s, acting only during the acute phase of the disease^6^. Nitazoxanide (NTZ) inhibits the enzyme pyruvate-ferredoxin oxidoreductase, disrupting the anaerobic energy metabolism essential for *Toxoplasma gondii* survival, which shows significant potential in the treatment of acute and chronic toxoplasmosis. This inhibition depletes the parasite energy without significantly affecting host cells due to the difference in metabolic pathways^7,8^.

The limitations of the traditional therapy for NTX have led studies to search for novel therapeutic alternatives to improve quality of life and adherence to treatment. Therefore, this study aimed to evaluate the efficacy of nitazoxanide in the *in vivo* treatment of NTX.

## MATERIALS AND METHODS

### Experimental animals and Me49 strain

*Swiss* 2-month-old female mice were obtained from the Animal Experimentation Vivarium at UFJ. The Me49 strain of *T. gondii* used in this study was provided by the IPTSP-UFG Laboratory of Parasite-Host Relationship. The strain was maintained by repeated inoculation in *Swiss* mice at the UFJ vivarium. After 60 days of infection, these animals were euthanized for brain extraction, obtaining the strain to use in the study^9^.

### Infection and experimental design

The infection of the study animals was carried out by gavage, by administering 10 cysts of the Me49 strain in 0.10 ml of 0.9% saline solution^10^.

### Experimental design

The animals in this study were divided into two groups (Group I and Group II), each subdivided into three subgroups, each subgroup consisting of four mice. Group I was subdivided into: I-A, composed of noninfected and untreated animals; I-B, composed of infected animals treated with 0.9% saline solution; and I-C, composed of infected and untreated animals. In Group II, all animals were infected and comprised the following subgroups: II-A, treated with oral suspension of NTZ 100 mg/kg/day (ANITTA^®^); II-B, treated with oral suspension of NTZ 150 mg/kg/day (ANITTA^®^); and II-C, treated with oral suspension of Pyrimethamine 4.4 mg/kg/day + Sulfadiazine 250 mg/kg/day. Treatment for each subgroup of animals began 30 days after infection, lasting 14 consecutive days.

After treatment, all mice were euthani*z*ed using an overdose of anesthetics and their kidneys, liver, and brain were removed for histopathological analysis. Moreover, blood from all animals was collected by cardiac puncture for biochemical analysis and cytokine level evaluation.

### Histopathological analysis and biochemical assay

Histological sections obtained from tissue samples of kidney, liver, and brain were stained with hematoxylin and eosin (H&E) staining for histopathological assessment under an optical microscope. The parameters evaluated included degrees of inflammation, edema, hyperemia, hemorrhage, necrosis, and vasculitis^11-13^. Inflammatory foci were evaluated throughout the histological section and inflammation was graded as mild, moderate, severe, and very severe, as described by El-Kowrany *et al*.^11^ and Bernardes *et al.* ^12^. Calcified cysts and the presence of cysts were evaluated according to the presence of degenerated, necrotic tissue and edema around the cyst, as well as morphologically, based on the loss of the tissue framework of the necrotic tissue, agglomeration of cellular debris due to the decrease in pH and, finally, precipitation of calcium on top of the degenerated tissue^14-17^.

Serum levels of aspartate aminotransferase (AST) and alanine aminotransferase (ALT), as well as urea and creatinine levels, were determined by biochemical assays following the manufacturers’ instructions, Bioclin^®^ and Laborlab^®^, respectively. Plasma samples from the animal groups were pooled for these biochemical assays due to the low quantity of material obtained individually from each animal.

### Cytokine quantification

Serum quantification of interleukin (IL)-2, IL-4, IL-6, interferon gamma (IFN-γ), tumor necrosis factor-alpha (TNF), IL-17A, and IL-10 was performed by flow cytometry. The Cytometric Bead Array (CBA) Mouse Th1/Th2/Th17 Cytokine Kit (BD Biosciences, USA^*®*^) was used for this analysis following the manufacturer’s recommendations.

### Statistical analysis

Analysis of histopathological results was performed using Chi-square and Mann-Whitney’s tests in the RStudio open-source software version 4.0.5. Differences were considered significant when p < 0.05.

Nonparametric tests, based on analysis of variance (ANOVA) and the Kruskal-Wallis test followed by the Dunn’s test, were employed for multiple comparisons of the means from three independent experiments related to the serum concentration of cytokines and biochemical factors. These analyses were performed in the RStudio open-source software version 4.0.5. A p-value < 0.05 was considered statistically significant.

### Ethical considerations

The study received approval from the Research Ethics Committee on Animal Use (CEUA) of the Federal University of Jatai (UFJ) under protocol Nº 006/2020.

## RESULTS

### Brain histopathological evaluation

**Figure 1:**
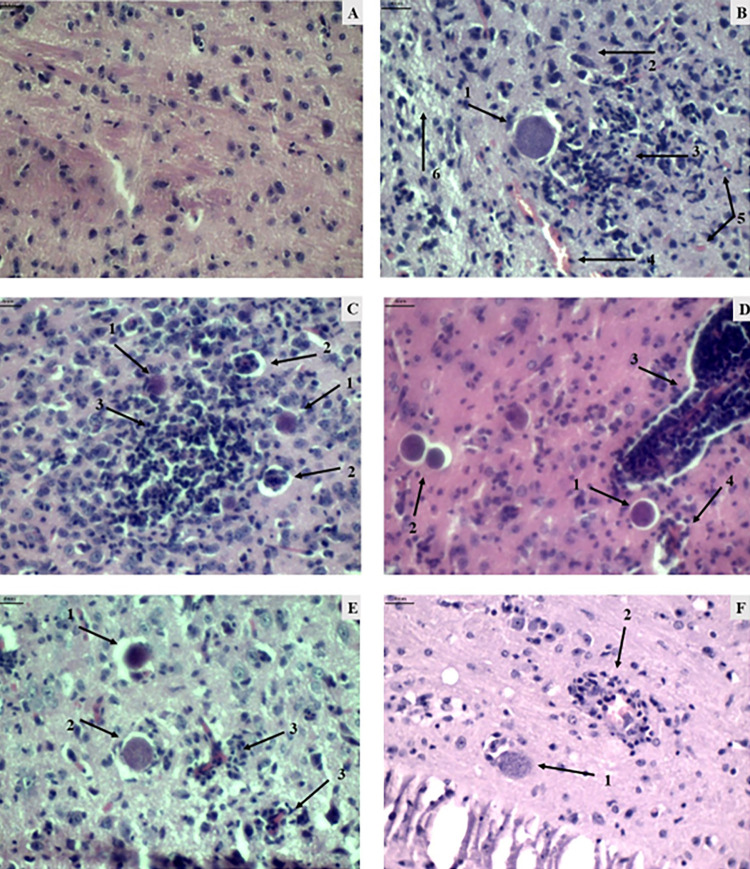
Histological photomicrographs of mouse brain tissue (magnification 400 times). A) Group I-A (uninfected and untreated): representation of brain tissue within the normal pattern. B) Group I-B (infected and saline-treated): presence of cysts (1), plasma cell (2), inflammatory infiltrate (3) both diffuse and focal, hyperemic vessel (4), hemorrhage (5), and edema (6). C) Group I-C (infected and untreated): cyst (1), vasculitis (2), focal (3), and diffuse inflammation (4). D) Group II-A (infected treated with NTZ 100mg/kg/day): cyst (1), calcified cyst (2), vasculitis (3), and hyperemia (4). E) Group II-B (infected treated with NTZ 150mg/kg/day): presence of cysts (1), presence of calcified cysts (2), and vasculitis (3). F) Group II-C (infected treated with Pyrimethamine and Sulfadiazine): presence of cyst (1) and vasculitis (2). Scale for all images = 4 nm.

Histological sections of the brain stained with H&E from different animal groups were analyzed 30 days after challenge to identify signs of inflammation and the presence of cysts. The parameters assessed to determine inflammation included the presence and quantity of infiltrates, signs of hyperemia, edema, bleeding, and necrosis (Figure 1).

The control group (I-A) did not show evidence of significant tissue injury and inflammation (data not shown), whereas all groups with infected animal, whether treated or not (Groups I-B, I-C, II-A, II-B, and II-C), exhibited histopathological alterations with varying degrees of inflammation and necrosis, without significant differences between them (Supplementary Tables S1, S2, and S3). However, the group of animals treated with NTZ 100 mg/kg/day or Pyrimethamine and Sulfadiazine (Groups II-A and II-C) showed the lowest incidence of hemorrhagic processes (Supplementary Tables S1 and S2), with a significant difference compared to the untreated group.

Regarding *T. gondii* cysts calcification, the 150 mg/kg /day group NTZ (II-B) exhibited a significantly higher frequency of cyst calcification among the infected groups, while the group Pyrimethamine and Sulfadiazine (II-C) showed the lowest frequency of cyst calcification (Supplementary Table S3). The pyrimethamine and sulfadiazine group (II-C) presented the lowest parasitic load among all treated and untreated groups.

### Hepatic histopathological evaluation

**Figure 2:**
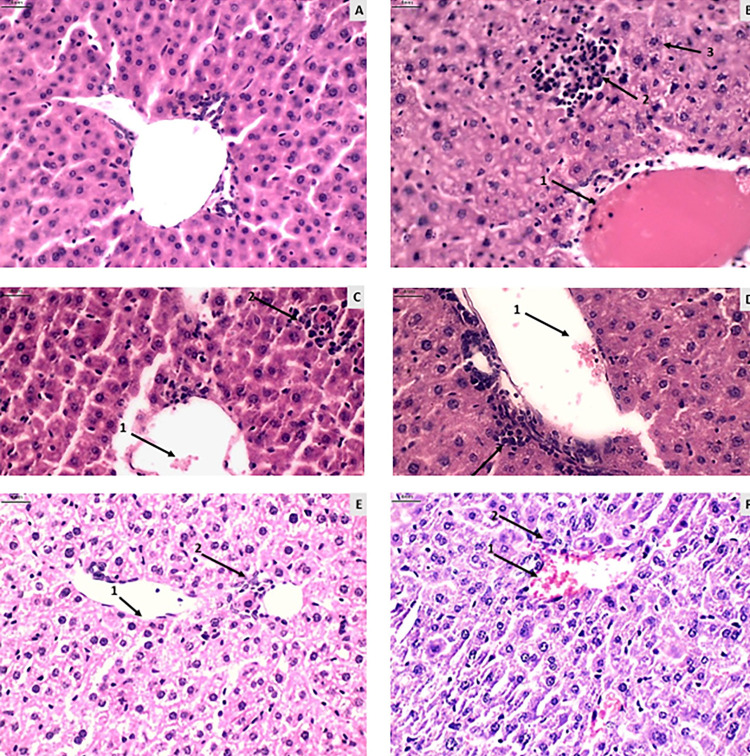
Histological photomicrographs of mouse liver tissue (magnification 400 times). A) Group I-A (uninfected and untreated): representation of liver tissue within the normal pattern with minimal hyperemia and inflammation. B) Group I-B (infected and saline-treated): presence of pronounced hyperemia (1), inflammation (2), and edema (3). C) Group I-C (infected and untreated): presence of hyperemia (1), inflammation, and necrosis (3). D) Group I-D (infected and untreated): presence of hyperemia (1) and inflammation (2). E) Group II-B (infected treated with NTZ 150mg/kg/day): presence of vasodilation (1) and inflammation (2). F) Group II-C (infected treated with Pyrimethamine and Sulfadiazine): presence of hyperemia (1) and inflammation (2). Scale for all images = 4 nm.

No significant differences were found in hyperemia, necrosis, and steatosis parameters between the evaluated groups. However, noticeable edema and significant moderate to severe liver inflammatory processes were observed among the groups treated with NTZ and untreated (Figure 2).

On the other hand, the Pyrimethamine and Sulfadiazine group (II-C) exhibited a higher prevalence of moderate liver inflammatory processes. Variable edema was more pronounced, with a significant difference in the NTZ treated groups compared to the untreated and Pyrimethamine and Sulfadiazine (II-C) groups (Supplementary Tables S4 and S5).

**Figure 3:**
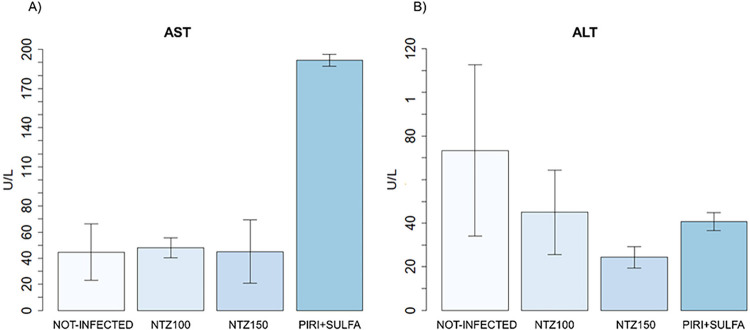
Serum concentration of AST and ALT enzymes in the study groups. Statistical analysis was performed using the Kruskal-Wallis test, followed by the Dunn’s test. Significant differences compared to the control subgroup and the treated subgroups are denoted by (* p < 0.05).

In Figure 3, the concentrations of the enzymes AST and ALT are depicted across the study groups. The AST/ALT concentration is higher in the Pyrimethamine and Sulfadiazine group (II-C) compared to both the control group and the NTZ treated infection groups.

### Renal evaluation

**Figure 4:**
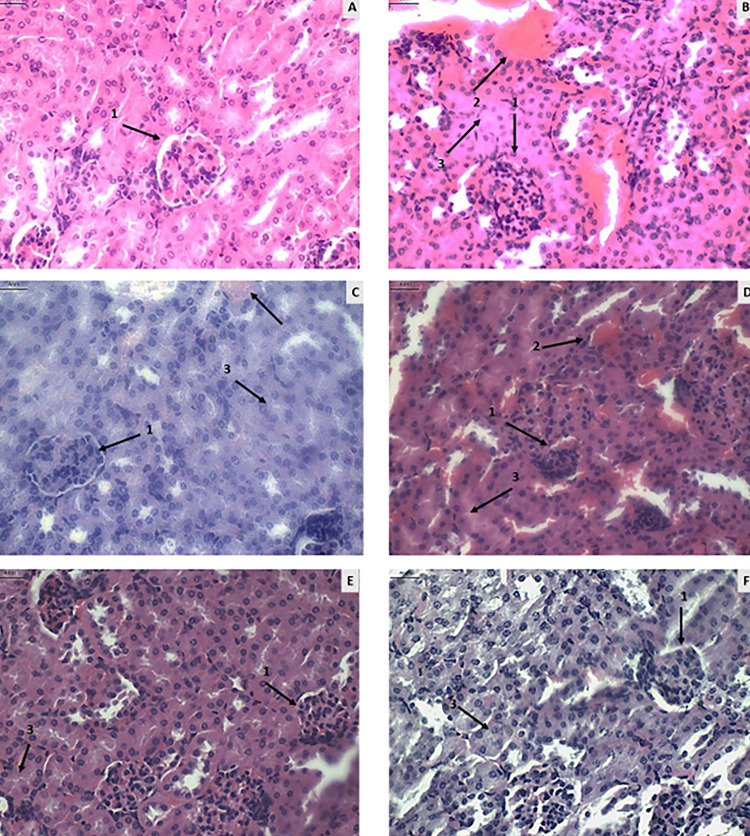
Histological photomicrographs of mouse kidney tissue (magnification 400 times). A) Group I-A (uninfected and untreated): representation of kidney tissue within the normal pattern with glomeruli showing no alteration of Bowman’s capsule (1). B) Group I-B (infected and saline-treated): presence of altered glomerulus with changes in Bowman’s capsule (1), pronounced hyperemia and vasculitis (2), and tubular alteration (3). C) Group I-C (infected and untreated): presence of glomerular alteration with reduced Bowman’s capsule (1), hyperemia and vasculitis (2), and tubular alteration (3). D) Group II-A (infected treated with NTZ 100mg/kg/day): presence of glomerular alteration with reduced Bowman’s capsule (1), hyperemia (2), and tubular alteration (3). E) Group II-B (infected treated with NTZ 150mg/kg/day): presence of glomerular alteration (1) and tubular alteration (3). F) Group II-C (infected treated with Pyrimethamine and Sulfadiazine): presence of glomerular alteration (1) and tubular alteration (3). Scale for all images = 4 nm.

Compared to the untreated and noninfected group (Supplementary Table S6), an increase in vasculitis and a presence of severe inflammation were observed in the groups treated with NTZ and Pyrimethamine and Sulfadiazine (II-C), together with severe glomerular and tubular alterations (Figure 4).

**Figure 5:**
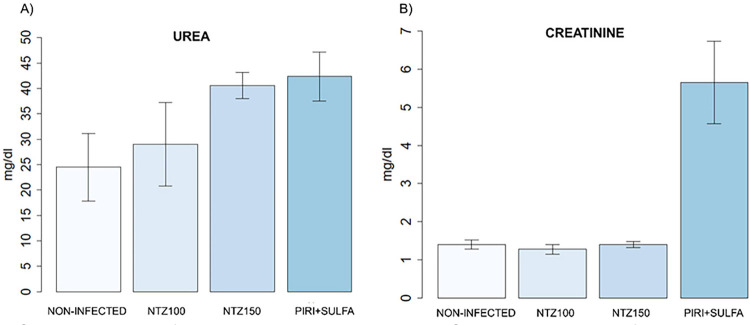
Serum concentration of urea and creatinine in the study groups. Statistical analysis was performed using the Kruskal-Wallis test, followed by the Dunn’s test. Significant differences compared to the control subgroup and the treatment subgroups are indicated by (* p < 0.05).

No significant differences were found in the biochemical assessment of the metabolic residues urea and creatinine between the evaluated groups (Figure 5).

### Cytokine quantification

Serum cytokine quantification (IL-2, IL-4, IL-6, IFN-γ, TNF, IL-10, and IL-17A) was performed on the 15^th^ day of treatment. No significant levels of IL-2 and IL-4 were detected by the methodology used. Infection with *T. gondii* increased the production of all other cytokines (IL-6, IFN-γ, TNF, IL-10, and IL-17A) in all experimental groups.

**Figure 6:**
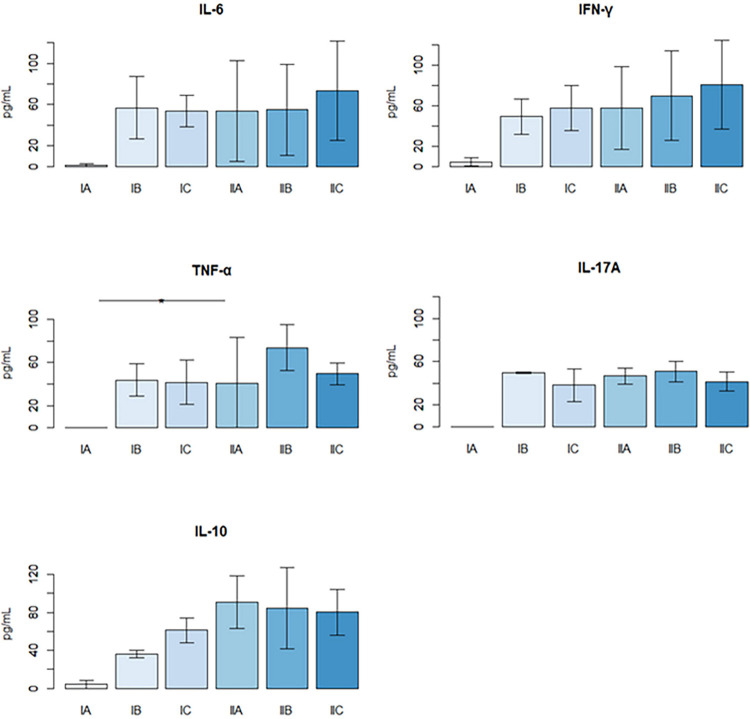
Serum cytokine quantification on the 15th day of treatment. Groups: I-A (uninfected and untreated), I-B (infected and treated with 0.9% saline), I-C (infected and untreated), II-A (infected treated with NTZ 100mg/kg/day), II-B (infected treated with NTZ 150mg/kg/day), and II-C (infected treated with Pyrimethamine and Sulfadiazine). The lines depicted in the figure represent the means of each group.

However, the difference between the levels of IL-6, IFN-γ, and IL-10 cytokines was not statistically significant. Only the concentration of TNF was significantly different between Group II-B (infected and treated with NTZ 150 mg/kg/day) and Group I-A (non-infected). Moreover, it should be noted that serum IL-10 levels are elevated in infected groups treated with NTZ 100 mg/kg/day and 150 mg/kg/day (Figure 6). However, none of the treatments significantly decreased the production of these cytokines compared to animals that were only infected and not treated.

## DISCUSSION

### Anti-toxoplasma action

Infection with *Toxoplasma gondii* resulted in the formation of inflammation, vasculitis, hyperemia, edema, and necrosis in all infected animals (Supplementary Tables S1 and S2), which was expected since these processes are typical responses to parasitic infection. We found that the groups treated with NTZ 100 mg/kg/day and Pyrimethamine and Sulfadiazine (Group II-C) exhibited significantly lower levels of hemorrhage in brain tissues compared to the infected and untreated control group^7^. This suggests that these drugs may reduce the perivascular inflammatory process^18,19^.

The group treated with NTZ 150 mg/kg/day showed a significantly higher frequency of calcified cysts compared to the group treated with pyrimethamine and sulfadiazine (II-C). This calcification suggests that NTZ at a higher concentration may stimulate the death of bradyzoites, leading to a reduction in cysts. Despite the Pyrimethamine and Sulfadiazine group presenting the lowest parasitic load (Supplementary Table S3), the results obtained with NTZ are also promising, indicating its potential as an alternative or complementary treatment for neurotoxoplasmosis.

In this context, *T. gondii* infection was observed to increase levels of IL-6, IFN-γ, and IL-10 in all experimental groups. A common mechanism to control *T. gondii* infection involves the production of pro-inflammatory substances, including innate immunity cytokines such as IL-6, IL-12, and TNF, and the adaptive immune response profile of Th1 lymphocytes, such as IFN-γ^20-23^. Deficient signaling of the cytokines IL-6, IL-12, TNF, and/or IFN-γ promotes excessive parasite growth, especially in acute cases^24-26^.

Moreover, to control an exaggerated inflammatory response, a concurrent anti-inflammatory response is essential. The cytokine IL-10 plays a crucial role in host survival by limiting the extent of the inflammatory response, thereby maintaining a balanced immune reaction^23-26^.

Although no significant differences were found in overall cytokine levels, this study found that NTZ treatment in animal groups effectively maintained parasite replication control. This was evidenced by a reduction in cysts and low parasitic load, while also regulating excessive inflammatory activity, which contributed to reduced animal mortality.

### Hepatic toxicity

The hepatic inflammatory processes observed in the groups treated with NTZ and Pyrimethamine and Sulfadiazine (II-C) (Supplementary Table S4) may be associated with drug toxicity, given that the liver is the primary organ responsible for metabolizing and inactivating substances^27^. Another factor that may be associated with this liver inflammatory process is *T. gondii* infection, as there are reports that this parasite can trigger liver injury and inflammation^27,28^.

The high levels of AST in the Pyrimethamine and Sulfadiazine group (II-C) may be related to various factors, as the AST enzyme is found in various tissues. A possible cause of this high level might be the inflammatory process observed in histopathological evaluation. Considering that the level of ALT in this group did not show a significant difference, it is inferred that this group did not show a hepatotoxic action, as ALT is a marker of liver injury with greater sensitivity and specificity than AST^29,30^.

We found no significant differences when comparing the histopathological results (Supplementary Table S5) and AST/ALT levels (Figure 2) between the Pyrimethamine and Sulfadiazine (II-C) and NTZ treated groups. This indicates that both conventional treatment and NTZ treatment showed similar results in hepatic toxicity.

### Renal toxicity

As observed in the renal histopathological evaluation, the groups treated with NTZ and Pyrimethamine and Sulfadiazine (II-C) showed, to some degree, alterations in Bowman’s capsule, tubular changes, vasculitis, and inflammation, suggesting possible renal damage. This damage may be associated with drug toxicity, as the kidneys filter, metabolize, and excrete most medications^31^. Another possible factor related to this renal injury is *T. gondii* infection, as identified by Pereira *et al*.^32^, who observed renal lesions in mice infected with the ME49 strain.

We found no significant differences when histopathological results were compared with urea and creatinine levels between the Pyrimethamine and Sulfadiazine (II-C) and NTZ treated groups. This indicates that both conventional treatment and NTZ treatment showed similar results in nephrotoxicity.

## CONCLUSIONS

Based on the toxicity analysis, it is evident that both NTZ and pyrimethamine associated with sulfadiazine exhibited similar levels of liver and kidney toxicity for the host. Among the concentrations of NTZ used, the 150 mg/kg/day concentration showed the most efficient anti-*Toxoplasma* action. Although the 150 mg/kg/day NTZ treated group did not achieve results as favorable as the Pyrimethamine and Sulfadiazine group (II-C) in controlling parasitic load, the results obtained suggest that NTZ shows significant potential as an alternative or complementary therapeutic regimen in the treatment of neurotoxoplasmosis.
